# Neonatal infrared thermography images in the hypothermic ruminant model: Anatomical-morphological-physiological aspects and mechanisms for thermoregulation

**DOI:** 10.3389/fvets.2022.963205

**Published:** 2022-08-04

**Authors:** Daniel Mota-Rojas, Dehua Wang, Cristiane Gonçalves Titto, Julio Martínez-Burnes, Dina Villanueva-García, Karina Lezama, Adriana Domínguez, Ismael Hernández-Avalos, Patricia Mora-Medina, Antonio Verduzco, Adriana Olmos-Hernández, Alejandro Casas, Daniela Rodríguez, Nancy José, Jennifer Rios, Alessandra Pelagalli

**Affiliations:** ^1^Neurophysiology, Behavior and Animal Welfare Assessment, DPAA, Universidad Autónoma Metropolitana (UAM), Unidad Xochimilco, Mexico City, Mexico; ^2^School of Life Sciences, Shandong University, Qingdao, China; ^3^Laboratório de Biometeorologia e Etologia, FZEA-USP, Faculdade de Zootecnia e Engenharia de Alimentos, Universidade de São Paulo, Pirassununga, Brazil; ^4^Animal Health Group, Facultad de Medicina Veterinaria y Zootecnia, Universidad Autónoma de Tamaulipas, Victoria City, Mexico; ^5^Division of Neonatology, National Institute of Health, Hospital Infantil de México Federico Gómez, Mexico City, Mexico; ^6^Facultad de Estudios Superiores Cuautitlán, Universidad Nacional Autónoma de Mexico (UNAM), Mexico City, Mexico; ^7^Division of Biotechnology—Bioterio and Experimental Surgery, Instituto Nacional de Rehabilitación-Luis Guillermo Ibarra Ibarra (INR-LGII), Mexico City, Mexico; ^8^Department of Advanced Biomedical Sciences, University of Napoli Federico II, Naples, Italy

**Keywords:** cattle, goat, newborn/neonate, sheep, water buffalo, wild ruminants, thermoregulation, infrared thermography

## Abstract

Hypothermia is one factor associated with mortality in newborn ruminants due to the drastic temperature change upon exposure to the extrauterine environment in the first hours after birth. Ruminants are precocial whose mechanisms for generating heat or preventing heat loss involve genetic characteristics, the degree of neurodevelopment at birth and environmental aspects. These elements combine to form a more efficient mechanism than those found in altricial species. Although the degree of neurodevelopment is an important advantage for these species, their greater mobility helps them to search for the udder and consume colostrum after birth. However, anatomical differences such as the distribution of adipose tissue or the presence of type II muscle fibers could lead to the understanding that these species use their energy resources more efficiently for heat production. The introduction of unconventional ruminant species, such as the water buffalo, has led to rethinking other characteristics like the skin thickness or the coat type that could intervene in the thermoregulation capacity of the newborn. Implementing tools to analyze species-specific characteristics that help prevent a critical decline in temperature is deemed a fundamental strategy for avoiding the adverse effects of a compromised thermoregulatory function. Although thermography is a non-invasive method to assess superficial temperature in several non-human animal species, in newborn ruminants there is limited information about its application, making it necessary to discuss the usefulness of this tool. This review aims to analyze the effects of hypothermia in newborn ruminants, their thermoregulation mechanisms that compensate for this condition, and the application of infrared thermography (IRT) to identify cases with hypothermia.

## Introduction

Hypothermia in newborn animals is caused by exposure to an ambient temperature significantly lower than that of the intrauterine environment (10–15°C lower than the core temperature) (1–3). The animal's body temperature is below the thermoneutral zone (4), which means heat loss exceeds heat production (5). This condition can affect the newborn's vitality by impeding the colostrum ingestion necessary to acquire the energy resources required to produce heat (6–9) and its importance lies in its impact on neonate mortality. Observed mortality rates in lambs are around 15%, while bovine calves range from 10 to 20% (10). Factors like the season of the year, environmental temperature (exposure to cold, rain, and wind) (11, 12), hypoxia and acidosis (in cases of dystocia), low birth weight, and inadequate nutrition of the mother during gestation are also elements that can induce hypothermia (3, 13, 14).

Unlike altricial species, precocial species, including ruminants, are more advanced neurodevelopmentally at birth. This provides them with highly efficient thermoregulatory mechanisms and an enhanced capacity to maintain homeothermy in diverse environments. The two main mechanisms are the vasomotor response and shivering or non-shivering thermogenesis (10). These mechanisms characterize the neonates of many animal species, but their nature and efficiency depend on the environment, species (precocial *vs*. altricial) (3), anatomical and physiological traits like coat type (hair, wool, fur), and the proportion of type II muscle fibers (15–17).

The physiological consequences and productive impact of hypothermia in newborns are potentially severe, so it is essential to recognize this condition promptly. Infrared thermography (IRT) has been proposed as a complementary, reliable, sensitive technique for assessing body temperature in animals (17, 18), but IRT's temperature recording in different anatomical regions has not yet been validated as an instrument for evaluating degrees of hypothermia. Moreover, available literature about the application of IRT in neonatal ruminants is required to determine its accurate application. This article aims to analyze the effects of hypothermia on newborn ruminants, their coping thermoregulatory mechanisms, and the usefulness of IRT for recognizing hypothermic conditions.

## Effect of hypothermia on newborn ruminants, a precocial model

Newborn ruminants are precocial animals with advanced locomotor development that allows them to stand up within the first minutes, or hours, of life and begin to ingest colostrum. They also have thermoregulatory mechanisms that allow them to maintain or reestablish normothermia (19), but several factors can affect them (3). Differences in heat production efficiency have been observed among species. For example, lambs are reported to have a higher maximum caloric rate per unit weight than bovine calves (20–22), a difference that helps explain why small ruminants like lambs or kid goats are more susceptible to hypothermia than larger ones [e.g., domestic bovine calves (*Bos taurus* or *Bos indicus*) or water buffaloes]. Also, a large surface area relative to mass shows a disadvantage in retain heat (23).

Activating the responses to prevent a drop in core temperature has physiological consequences (1, 7, 24, 25), especially when hypothermia persists for extended periods because it triggers vasoconstriction as a circulatory reaction that prevents heat loss. However, vasoconstriction has adverse effects, such as decreasing blood flow to high-demand tissues in the neonate, and inducing cellular hypoxia (26). Moreover, in animal models at the histological level, even cerebral neuronal necrosis (histopathological hypothermia score = 2 vs. control = 0) has been reported by Petko et al. (27). Hypoxia also has metabolic consequences. Tyler and Ramsey (28) evaluated the effect of reduced blood oxygen in 12 Holstein calves at 72 h postpartum. They found that due to hypoxia, CO_2_ levels did not exceed 45 mmHg, and glucose concentrations tended to decline in the hypoxic calves (110 vs. 105 mg/dl), while lactate levels increased between 6 and 42 h postpartum to values of 90 mg/dl in hypoxia group and 23 mg/dl in the control group. In a 28 blue and white Belgian calves' study, Cambier et al. (29) analyzed blood oxygen transport and tissue oxygenation. In hypoxic calves, acidosis and hypercapnia were the main effects caused by decreased oxygen affinity for hemoglobin in arterial blood, which was related to the amount of O_2_ in the bloodstream (control group = 77.2 ± 9.9 mmHg vs. hypoxemic group = 47.9 ± 12.9 mmHg). According to those results, hypoxia caused the organisms to utilize anaerobic metabolic pathways that triggered lactate production, which induced a state of acidosis and hypoglycemia that reduced the animal's capacity for thermogenesis (21, 22, 30).

In response to the decreased oxygen supply to tissues, an increase in air volume in ventilation occurs with each inspiration that can produce tachycardia in animals to improve oxygen distribution (31). The increase in cardiac output and the production of erythrocytes are other compensatory responses to improve oxygen transport and nutrient delivery to tissues (26). These physiological consequences become more pronounced when the mechanisms cannot operate efficiently because the neonate has limited energy reserves (such as glucose and oxygen). This condition can arise, for example, when a newborn consumes and may severely deplete those reserves for purposes of thermoregulation during the first 12 h post-birth.

The ability of young ruminants to stand up and ingest colostrum in the first hours of extrauterine life allows them to acquire energy and maintain a thermoneutral state (3, 32). Nutrition in newborns has been discussed in depth by authors like Hammon et al. (33), who sustain that colostrum provides nutrients that include glucose, a biomolecule used to combat hypothermia. The supply of immunoglobulins through colostrum, and their absorption, are other elements that help maintain homeostasis in neonates. As mentioned above, acidosis can be an adverse effect of hypoxia derived from hypothermia. Some reports on bovine calves indicate that a blood pH of 7–7.20 and high pCO_2_ concentrations (respiratory acidosis) are negatively related to immunoglobulin G1 absorption (IgG) (34). However, a study of 48 Holstein calves found no association between the degree of IgG absorption and increased PaCO_2_ concentrations for 1 h (35). Therefore, it has been suggested that hypothermia generates metabolic consequences and affects newborns' underdeveloped characteristic immunological system.

Likewise, environmental factors such as the year's season may expose newborn ruminants to low temperatures. For example, for species like the water buffalo, there are reports of high mortality rates in autumn, when cold and rain predominate (36). However, this relation has not been specifically evaluated because incidences of stillbirths can be affected by both environmental and genetic factors. It has been suggested that backcrossing may help diminish mortality rates in newborns (37). Moreover, anatomical and morphological characteristics can impact the physiological effects of hypothermia in newborns or the animals' adaptive measures to prevent such consequences.

## Anatomical-physiological characteristics of thermoregulation in ruminants

In ruminants, the degree of development at birth is considered similar within each species, though differences in rates of brain maturation may occur, as has been seen between lambs, which reach the maximum weight of cerebral parenchyma 50 days after birth, and kid goats where this occurs at just 20 days post-birth (38). Studies of sheep have shown that they lack awareness during the fetal stage and that the onset of this condition comes within 20 min post-birth. At that moment, these animals show voluntary responses or the involvement of the cerebral cortex in perceiving stimuli (39). The ensuing behavioral responses are recognized by mother-young interaction at 20–60 min post-birth. This time is called the sensitive period when newborns show a strong preference for their biological dams up to 24 h (40). Their interaction includes grooming, a behavior that promotes bonding and plays a role in thermoregulation as the dam cleans and dries the neonate's coat, thus reducing the amount of heat loss due to evaporation (41).

Another mechanism that helps prevent heat loss in neonatal precocial species is developing the locomotory system that allows newborns to stand up in the first minutes postpartum. Regueiro et al. (42) reported that newborn lambs stand up after an average of 22 min, and 53.9–87.9% began to suckle within 1 h of birth. Results for Australian Bush goat kids are similar, as they begin to stand at an average of 20.2 min and suckle at 50.8 min (43). Although neurological development is essential for maintaining the temperature of newborns, anatomical-physiological aspects are crucially involved in the thermogenic mechanisms that prevent heat loss, such as vasomotor control and heat production by contraction of the skeletal striated muscle or through the consumption of high-energy adipose tissue (1, 44).

In the first case, involuntary rhythmic contractions of skeletal striated muscle reflect the ratio of muscle fibers. The importance of a specific muscle fiber ratio lies in the degree of resistance and maintenance of the muscle contractions that can be produced. This has been proposed as a difference that may influence the efficiency of shivering thermogenesis, complemented by the individual morphology, percentage of body fat, diet and the degree of cold acclimatization (45). Recruitment of type I (dependent on aerobic metabolism) or slow-oxidative muscle fibers is associated with low-threshold (4–8 Hz), continuous shivering, and lipid consumption. In contrast, type II (fast-glycolytic) fibers respond to high-intensity bursts (0.1–0.2 Hz), produce burst shivering, and are specialized for carbohydrate utilization (46). Additionally, oxidative type I fibers show greater contractile efficiency than type II fibers, and their deeper location facilitates access to the bloodstream and a more efficient oxidative metabolism, unlike type II fibers, which are predominantly superficial (47). Type II fibers, called anaerobic muscles, play a critical role in exposure to cold environments because their glycolic fibers can be activated at a lower metabolic rate (45). Therefore, a high proportion of type II muscle fibers in ruminant neonates' first days of life can be considered an advantage due to their glycolytic metabolism and ATPase activity (48).

Because of their thermogenic characteristics, type II muscle fibers are more important for neonates. In Peinado et al.'s study of sheep (49), the percentage of type I and II fibers were similar on days 1–15 post-birth, but at 30 days, the percentage of type II fibers had decreased by 80%. Yates et al. (50) found that intrauterine growth restriction in seven fetuses of Columbia-Rambouillet ewes near the end of gestation reduced the proportion of type I muscle fibers. Their results showed that, in general, growth-restricted fetuses were 54% smaller and had lower blood glucose (0.69 ± 0.09 mM) and oxygen (2.15 ± 0.21 mM) levels than control animals (glucose= 1.05 ± 0.10 mM and O_2_= 3.40 ± 0.23 mM). Regarding the semitendinosus and biceps femoris muscles, the authors reported a lower ratio of type I to type II/IIa fibers than controls, which correlated negatively with epinephrine concentrations (*r* = −0.64) but positively with insulin levels (*r* = 0.62). These results confirm that in the neonatal stage, metabolically active muscle tissue contraction participates primarily in thermogenesis since it contains high energy reserves (e.g., glucose) that are available to generate heat.

Glucose is a valuable energy reserve in newborn ruminants because skeletal muscle glucose is less abundant in these species due to the presence of deposits of brown adipose tissue (BAT), in contrast to animals like piglets (51). Dühlmeier et al. (52) compared glucose use in four skeletal muscle samples from five kids and nine adult goats at different stages of life. They found that GLUT 4 glucose transporter expression was significantly lower in the muscle of feeding kids than in adults and differed in each stage of life in the latter.

The placenta maintains fetal glucose levels during gestation, but at birth and during lactation newborns rely on glycogen stores in the liver to maintain adequate plasma concentrations (51). This led to the interpretation that newborn ruminants consume hepatic glycogen as the primary energy source for heat production (53). For example, in ovine fetuses, glycogen can be observed in small amounts in BAT, but the amount of glycogen at birth is markedly reduced (54). This limit on glycogen storage in the liver and muscles at birth means that goat kids, lambs, and calves have low glucose concentrations that make them susceptible to hypoglycemia since those limited reserves can be depleted rapidly within the first 12 h postpartum. This effect exacerbates if the onset of feeding is delayed (55).

Hypoglycemia at birth has been reported in 22.5% of goat kids, but figures are significantly higher for the Sahel breed, twins, and newborns with low birth weight (55). When neonates' glucose concentrations are inadequate, they begin to consume muscular reserves through the Cori cycle. Specifically, type II fibers can conduct glycogen, so a high proportion is critical for thermoregulation. Therefore, glycogen availability is essential for newborn ruminants since calves or lambs require an elevated metabolic rate to control their temperature within the first hours of life (56).

Adipose tissue regulates temperature and energy balance through its metabolic and cellular functions. In mammals, this tissue is divided into white (WAT, anabolic function) and brown forms (BAT, catabolic function) (57, 58). BAT develops in the fetal stage (59). At birth, it constitutes a key element for non-shivering thermogenesis (7, 60) because it contributes to the survival of neonate ruminants exposed to cold temperatures (61). It has been suggested that BAT has similarities to WAT. However, significant differences at the histological level include uncoupling proteins 1 (UCP1), a central nucleus, multilocular triglyceride vacuoles, abundant mitochondria with dense parallel cristae, high vascularization, and a rich cytochrome content, elements that explain its brown color (62, 63). The characteristics of these elements can change with the age of newborns. Observations of goat kids at 4 days of age showed that lipid vacuoles increased in size but decreased in quantity, while at seven days, only a single central lipid vacuole and a nucleus and cytoplasm at the periphery were found, with the appearance of univacuolar WAT (64). A similar effect has been reported in 2-day-old Merino lambs, where the proportion of large local fat increased, but there were no small locules at 32 days of age (54).

**Figure 1:**
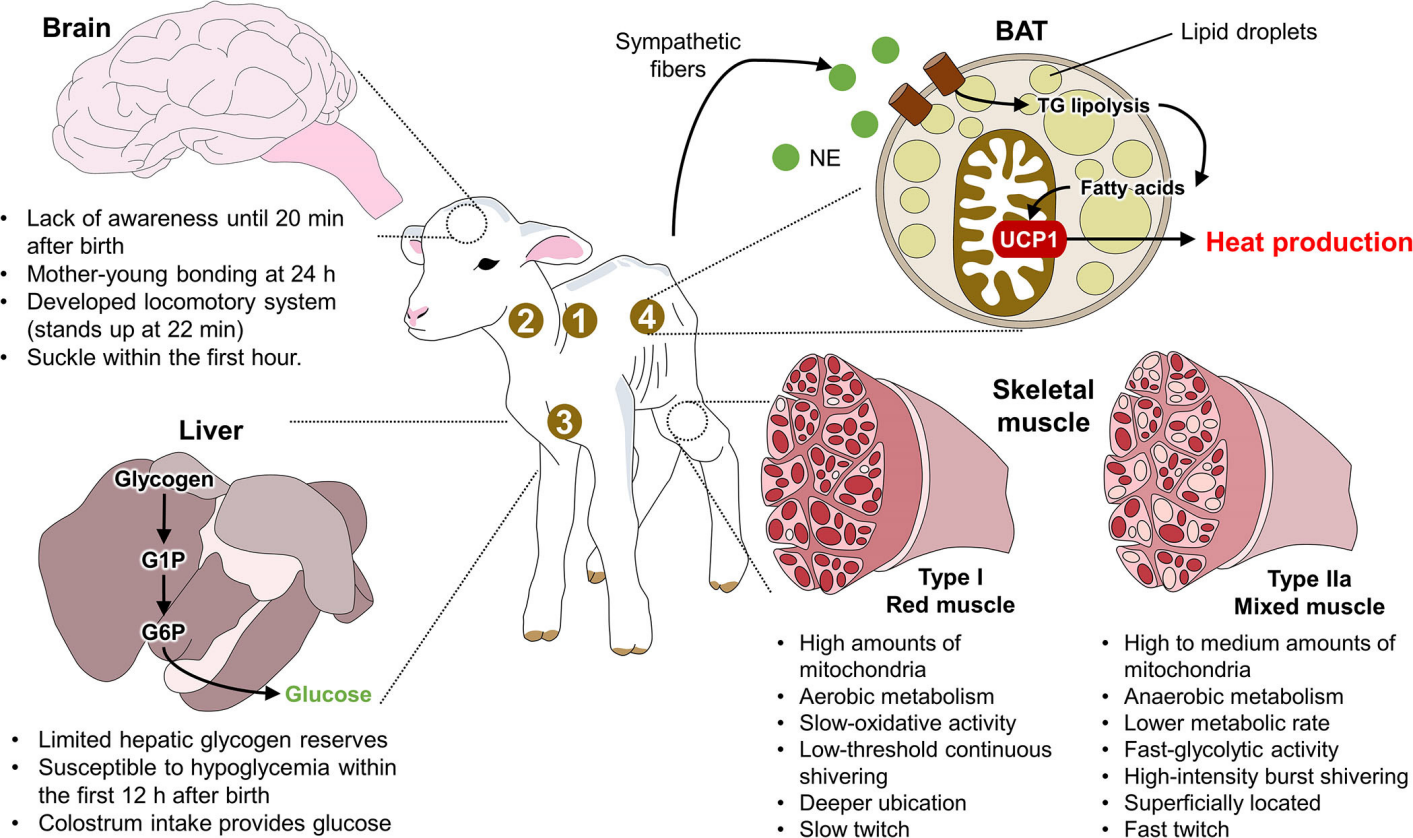
Anatomical traits of newborn ruminants. 1. supraclavicular region; 2. neck region; 3. pericardial region; 4. perirenal region; BAT, brown adipose tissue; G1P, glucose 1 phosphate; G6P, glucose 6 phosphate; NE, norepinephrine; TG, triglycerides; UCP1, uncoupling protein 1.

Although BAT is present in all ruminants, its distribution differs among species. For example, in goat kids, BAT pads are more common in the perirenal and inguinal regions (21, 64, 65). Studies of newborn piglets have shown a lack of WAT deposits, as those animals rely on BAT to produce heat in the main location sites, including the supraclavicular, neck, pericardial, and perirenal regions (65). Similarly, in wild ruminants like reindeer (*Rangifer tarandus* L), BAT is located mainly in the perirenal-abdominal region (66). In cattle, BAT is observed in the perirenal, subscapular, and retroperitoneal regions (59), and electron microscopy has shown that most of the adipose tissue in newborn calves is BAT. It represents 2% of body weight but is converted progressively into WAT during the first days of life (67). These differences among species in the location of BAT deposits suggest that degrees of thermogenetic efficiency depend on the amount and distribution of BAT and that a low amount of BAT increases susceptibility to hypothermia (13). Figure 1 summarizes the main neuroanatomical characteristics of newborn ruminants. In this figure, the lamb is used as an animal model to show the thermoregulatory characteristics of newborn ruminants, such as neurodevelopment and the limited hepatic glycogen reserves that make them susceptible to hypoglycemia. Specifically, the presence of brown adipose tissue (BAT) in the regions marked with numbers and the activity of type IIa muscle fibers function as coping mechanisms to produce heat by shivering and non-shivering thermogenesis.

Evidently, anatomical physiological differences among newborn ruminants constitute the physiological basis for the various thermoregulation mechanisms found in different ruminant species and contrast to those that characterize altricial species.

## Thermoregulation mechanisms to combat hypothermia in ruminants

The thermoregulatory responses of precocial species encompass physiological, metabolic, and behavioral mechanisms that depend on the activation of the central and autonomic nervous systems. The goal is to achieve thermogenesis by activating BAT and shivering. The vasomotor response that constricts the blood vessels prevents heat loss by signaling the thalamus to the lateral parabrachial nucleus (68). Related thermoregulatory processes are explained in greater detail below.

### Central and peripheral vasomotor control

Newborns exposed to cold suffer a drastic drop in core temperature that may exceed the body's ability to produce heat (5). The organism induces various responses to a marked deviation from the zone of thermoneutrality, activating immediate mechanisms to prevent heat loss through radiation.

One of these is the vasomotor mechanism, which changes the caliber of the superficial blood capillaries in the skin to prevent heat exchange with the surrounding medium (69–71). In this case, upon exposure to cold, sympathetic nerve fibers act on α2-adrenergic receptors to generate a vasomotor response that causes smooth muscle vasoconstriction of the superficial blood vessels (72). A study by Bligh et al. (73) used sheep and goats as models to study the effect of intraventricular injections of norepinephrine and acetylcholine on thermoregulatory performance and environmental temperature. They reported that administering norepinephrine lowered the respiratory rate and decreased body temperature when the animals were exposed to low ambient temperatures. In contrast, acetylcholine administration increased body temperature despite the low ambient temperatures. These results enhance our understanding that catecholamine neurosecretion tends to decrease heat loss in response to cold thermal signals as an immediate mechanism.

However, other results suggest that several factors can inhibit this mechanism, including the offspring's birth weight (74) and available energy resources (32). Studies have shown that newborns with low birth weight may have a lower average temperature, while those with higher birth weights do not exhibit the phenomenon of vasoconstriction during the first 12 h after birth (74). This may occur because vasomotor control is immature in animals with low birth weight and cannot function to reduce heat loss (75).

Similarly, the availability of energy resources at birth, such as colostrum, may provide the nutrients required to maintain heat production and compensate for low environmental temperatures (32, 76, 77). Caldara et al. (78) studied the effect of birth weight on body temperature in piglets. After recording the surface temperature of the animals using IRT and the humidity and temperature of the ambient air at 15, 30, 45, 60, and 120 min after birth, they found that surface temperature decreased at 15 min (32.55°C) post-birth but presented an upwards tendency at 30 min (35.05°C). Although a high negative correlation was recorded between humidity and the animals' surface temperature (*r* = −0.82 and −0.81) 15 min after birth, the weight of the piglets at birth presented a positive correlation with surface temperature (*r* = 0.47).

**Figure 2:**
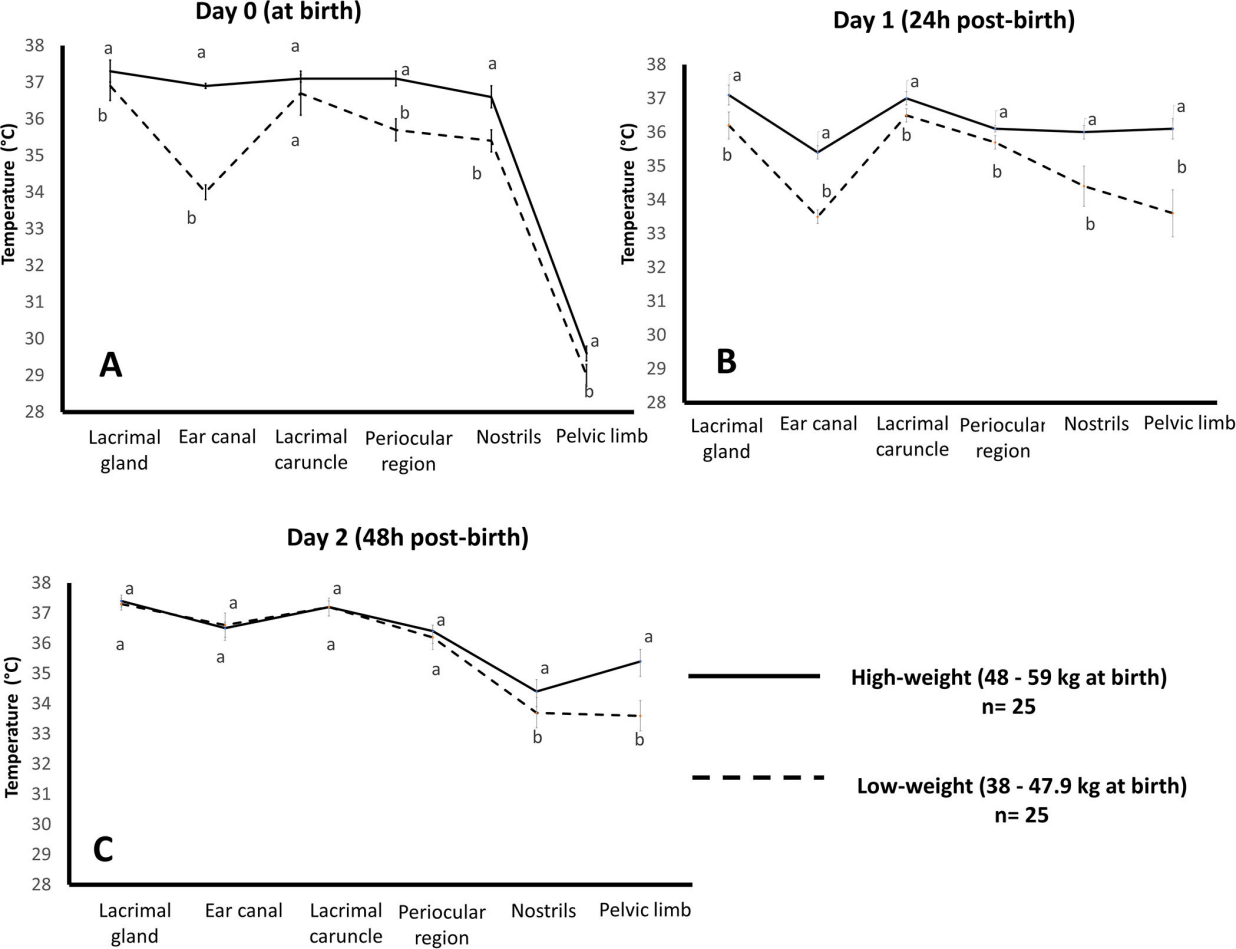
Effect of birth weight on the average temperature of water buffalo neonates (*n* = 50) in the first 48 h of life by evaluating six thermal windows. Data was obtained using variance analysis (ANOVA) by the mixed general linear model, with multiple comparisons of means using Holm-Sidak's test, Shapiro-Wilk normality test, and a significance level of *p* < 0.05. ^a, b^ Different literals indicate significant differences between groups of high- and low-weight buffalo calves (*p* < 0.05). Images were taken with a FLIR camera (FLIR Systems, Boston, MA, USA), 18 mm lens FOL, the emissivity of 0.95, IR resolution of 320 × 240, and a reflecting temperature of 20°C. The thermal images were processed by the software FLIR tools. **(A)** comparison of the mean temperature at parturition in groups with high and low birth weights, **(B)** comparison of the mean temperature at 24 h after parturition in groups with high and low birth weights, and **(C)** comparison of the mean temperature at 48 h after parturition in groups with high and low birth weights.

These results could be associated with those observed by our research team in a preliminary study of newborn water buffaloes (*Bubalus bubalis*) (*n* = 50), where the relation between birth weight (high birth weight: 48–59 kg *vs*. low birth weight: 38–47.9 kg) and surface temperature during the first 48 h of life was monitored in six thermal windows (ear canal, lacrimal gland, lacrimal caruncle, periocular region, nostrils, pelvic limbs). Figure 2 presents the thermal response of buffalo calves where (A) shows the comparison of the mean temperature at parturition in groups with high and low birth weights. At birth, results indicated that the group of high birth weight buffaloes at parturition had an average range of temperature of 0.4 to 2.9°C higher than that of the low-birth-weight group in all thermal windows, with statistically significant differences (*p* < 0.0001), except for the lacrimal caruncle. (B) Comparison of the mean temperature at 24 h after parturition in groups with high and low birth weights. At 24 h post-birth, both groups showed statistically significant differences (*p* < 0.0001) in all thermal windows. The high-weight group had a mean temperature of 0.4–2.5°C above the low-weight group. (C) Comparison of the mean temperature at 48 h after parturition in groups with high and low birth weights. At 48 h post-birth, significant differences (*p* < 0.0001) were only found in the nostrils and pelvic limb thermal windows, with an average difference of 0.7°C and 1.8°C, respectively. The effect that birth weight exerts on the thermoregulatory capacity of newborns is due to the limited capacity that low-birth-weight calves have (79). This is associated with newborns' fat reserves and body mass index thermoregulatory capacity (80). In lambs, low weight at birth is also accompanied by hypoglycemia (3), restricting their energy resources to produce and maintain corporal heat. In addition to this, low birth weight can impact the health and vitality of newborns (81). Another relevant issue is the immaturity of the skeletal muscle in newborns, so shivering thermoregulation is not an effective mechanism in these cases to produce the required heat (82). Therefore, reducing the superficial temperature of the present buffalo calves reflects a vasomotor mechanism to prevent heat loss by vasoconstriction.

These findings indicate that the vasomotor mechanism constitutes an immediate compensation response triggered to prevent greater heat loss through exchange with the environment. It is important to note that the compensatory response of the vasomotor tone has a limit. It may prevent all sensible heat losses in the newborn while the organism initiates additional responses or thermoregulatory mechanisms that require greater energy expenditure.

### Shivering thermogenesis

Shivering thermogenesis is the facultative thermogenic mechanism that produces heat at a resting level through active muscle contraction (1, 83–85). Some authors suggest that this ability is innate in precocial animals due to their degree of development at birth, but others, like Vanden Hole et al. (86), analyzed the level of movement and muscle contraction in newborn piglets in an attempt to show whether this is innate or matures rapidly in those animals. They evaluated gait patterns by analyzing spatial-temporal characteristics (stride frequency, step length, work factor) and left-right asymmetry step-by-step during gait in a corridor at different moments (0–96 h post-birth). Observations showed that the animals at 2 h of age had normalized values for the space-time variables, while asymmetry of the steps was around 10% within 8 h of birth. The authors affirmed that this means that the coordination of movements is not entirely innate in precocial animals but that a rapid neuromotor maturation occurs.

**Figure 3:**
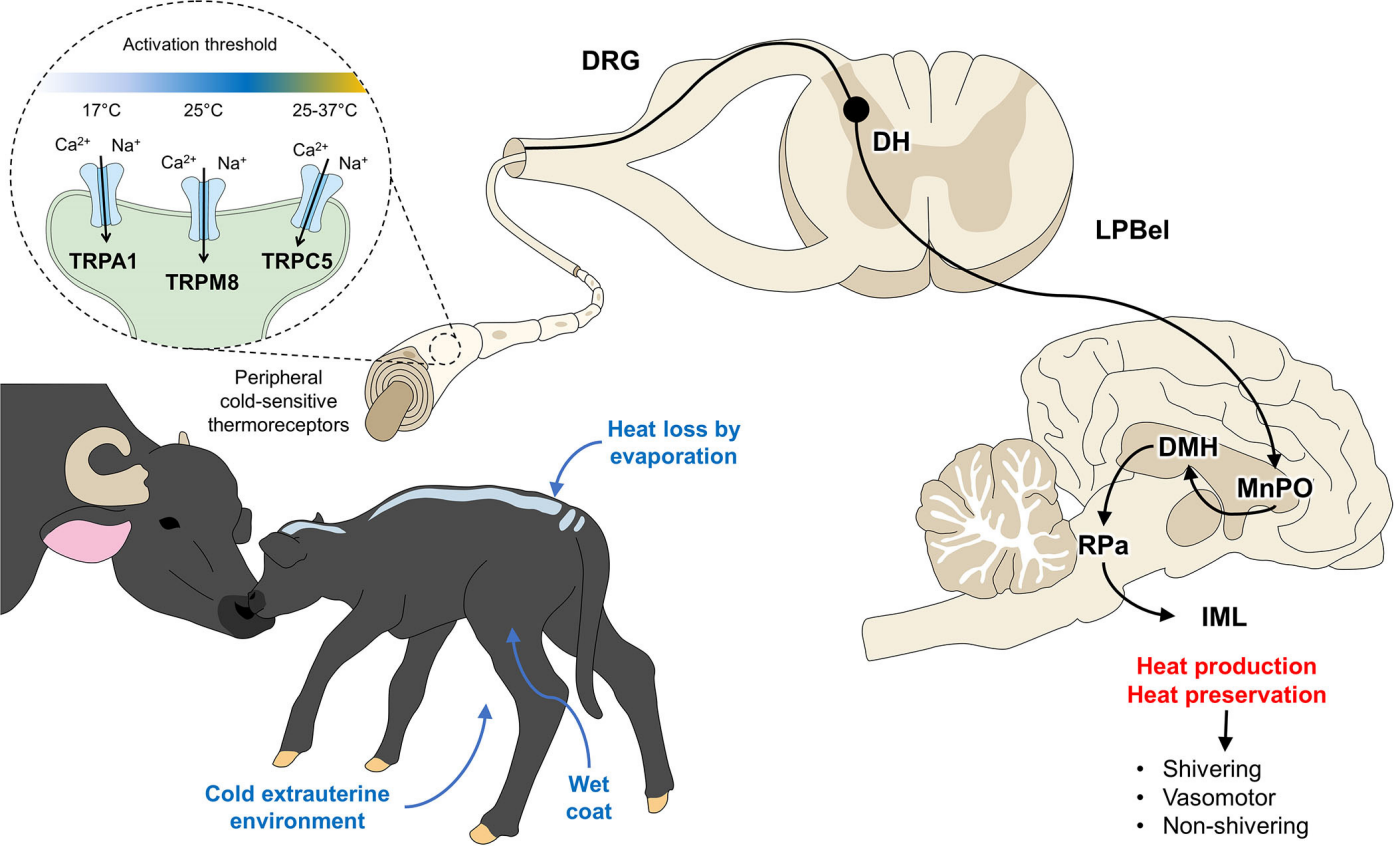
Hypothalamic response to hypothermia. Ca^2+^, calcium; DH, dorsal horn; DRG, dorsal root ganglion; IML, intermediolateral column; LPBel, external lateral part of the lateral parabrachial nucleus; MnPO, median preoptic nucleus; Na^+^, sodium; RPa, raphe pallidus. TRPA, transient receptor potential ankyrin 1; TRPC5, transient receptor potential canonical 5; TRPM8, transient receptor potential melastatin 8.

The neurophysiological shivering response has been described by researchers like Morrison and Nakamura (87) (Cited by Mota-Rojas et al.) (1), who described that the neurons of the dorsomedial hypothalamic nucleus (DMH) are activated in response to cold. These neurons allow the stimulation of neurons at the level of the raphe pallidus area (RPa) (88), which sends a signal to the sympathetic preganglionic neurons in the intermediolateral cell column (IML) that increases contraction at the muscular level to promote heat production by increasing the tremor amplitude of vibrations (89). This event is schematized in Figure 3. External factors such as a cold extrauterine environment, wet coat, and the consequent heat loss due to evaporation activate peripheral cold-sensitive thermoreceptors. These receptors transmit signals to the spinal cord and such supraspinal structures as the MnPO, the main thermoregulatory center. Its projections to the DMH, RPa, and IML in the spinal cord integrate mechanisms that generate or preserve heat through shivering or non-shivering thermogenesis and vasomotor changes. Although the importance of shivering thermogenesis is evident, reports on newborn lambs suggest that around 50% of heat production comes from shivering thermogenesis and 50% from non-shivering thermogenesis (90). This may be mainly attributable to the anatomical magnitude of the newborn, that is, the degree of muscle development (84, 91).

The degree of muscle development depends on the mother's diet during pregnancy, her percentage of fat, and its distribution according to breed. For example, Hereford calves have been reported to have higher fat content than Simmental calves at 2 days of age (92). In this context, Lammoglia et al. (93) studied the effect of supplemental fat feeding in pregnant female bovines during the last 55 days of gestation on the cold tolerance of F2 calves (Piedmontese and Hereford), which 5 h after delivery were exposed to a temperature of 0°C for 140 min. The prepartum mothers had received diets containing 2.2% (low-fat) or 5.1% fat (high fat). Findings showed that glucose levels were higher in the offspring of the mothers that had received the high-fat diet and that rectal temperatures were 0.5°C higher in those animals than in the neonates of the low-fat diet mothers. The authors commented that although the mother's diet is important for improving offspring survival, the degree of muscular development also plays a key role because it enhances the proportion of thermogenesis through shivering.

A similar study design by Dietz et al. (94) evaluated pregnant fall-calving heifers fed diets with different fat levels (1.5, 4, and 5%). The calves were exposed to a temperature of 5°C for 90 min. Observations showed that the responses to cold and the frequency of shivering in the newborns were similar despite the variable levels of energy administered. The provision of energy resources is a fundamental factor for the newborns' success in temperature compensation but may be a limited resource given the high energy cost it represents for the animals. The authors also mentioned that the muscular contraction response could occur only for a short time when animals are exposed to a temperature of 10°C (14).

Shivering thermogenesis is a compensatory response that is efficient in the short term, but factors like the degree of muscle development and maternal nutrition during gestation may influence neonates' resistance to cold.

### Non-shivering thermogenesis

**Figure 4:**
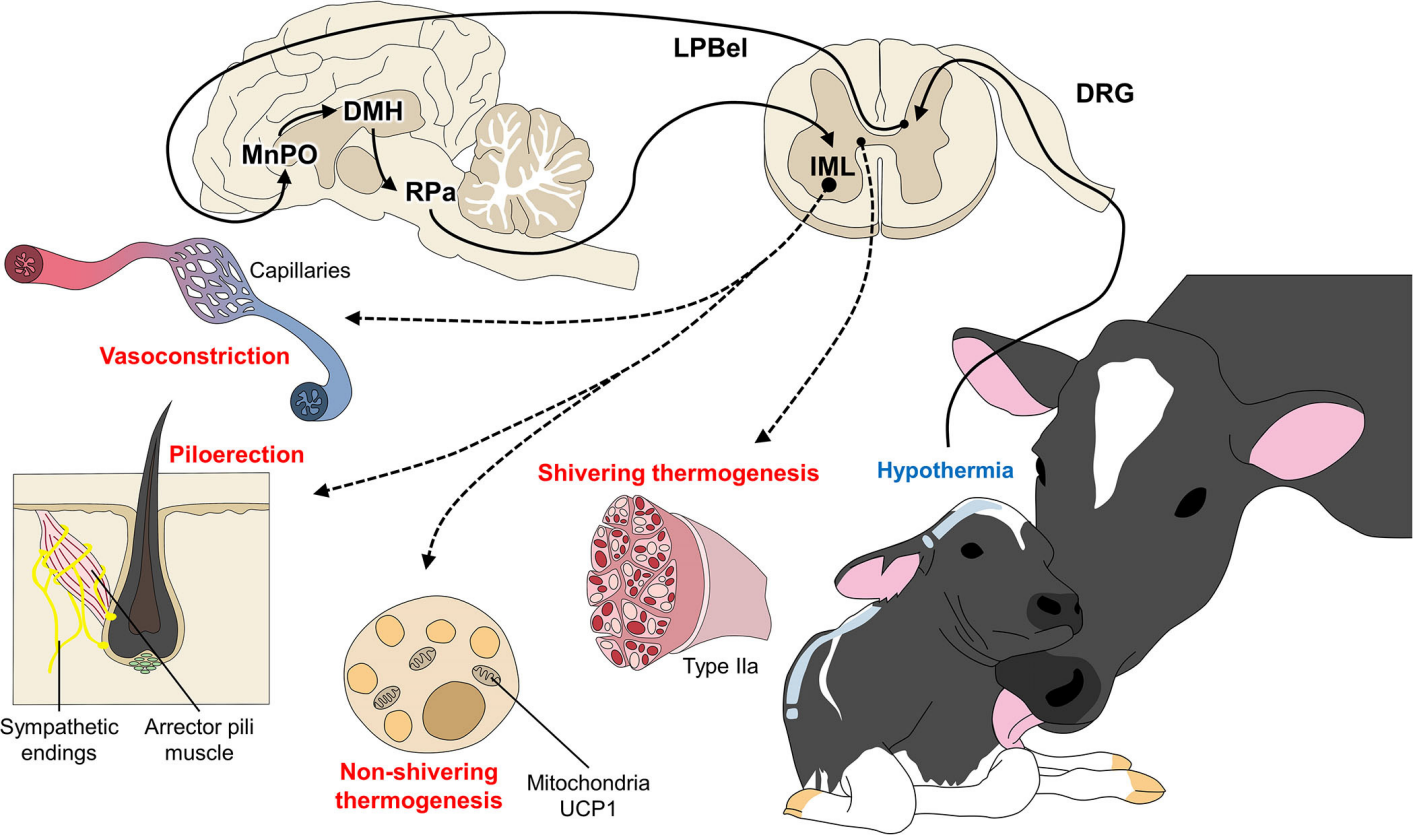
Peripheral responses as coping mechanisms for hypothermia. When the thermoregulatory center of the central nervous system (MnPO) perceives a decrease in body temperature, the organism of newborn ruminants (and other mammals) activates peripheral responses with two main objectives: **(A)** to prevent heat loss through vasoconstriction and piloerection; and **(B)** to produce heat by shivering and non-shivering thermogenesis, where BAT and skeletal muscle fibers participate in the return to a state of homeothermy.

Under hypothermic conditions, animals use NST as a short-term response, but if the condition is not overcome quickly, they may attempt to generate heat by consuming BAT (95, 96). This mechanism is called NST (97) and is critical for determining the offspring's metabolic adaptation to cold temperatures in the extrauterine environment (98–100). BAT is rich in energy cells that allow efficient heat production through sympathetic innervation (62, 68), which regulates the production of metabolic heat and vasomotor and piloerection responses to hypothermia (Figure 4).

Physiologically, BAT is activated by the release of norepinephrine from the nerve terminals of the sympathetic nervous system. Its thermogenic capacity is mediated by uncoupling protein-1 (UCP-1), which is found only in the mitochondrial adipocytes (1, 61, 65, 101). For this reason, BAT's heat producing-capacity is determined by the concentration of UCP-1 and the activity of the proton conductance pathway through which the electron transport chain uncouples the synthesis of adenosine triphosphate. The sympathetic fibers in BAT promote neurosecretion of catecholamines that stimulates the oxidation of fatty acids and the gene oxidation of UCP-1 for heat production under conditions of exposure to cold (101). These facts confirm the importance of food consumption during the first hours of life, the fact that newborns who are food-deprived for any reason have a limited ability to maintain their body temperature, and the importance of colostrum consumption.

Another factor that intervenes in thermogenesis is birth weight since low-weight animals may not have the energy resources necessary for heat production. As Dwyer (102) concluded in a study of the influence of birth weight on postnatal mortality in lambs, low birth weight animals had lower vitality scores, though they spent more time standing up. The fact that the act of standing up consumes abundant energy resources suggests that, in precocial animals, food ingestion can help maintain stable thermoregulation.

Colostrum is considered one of the main energy sources for newborns, and its thermogenic effect is associated with the production of metabolic heat through such processes as the digestion, absorption, and processing of nutrients. Moreover, as a source of lactose, amino acids, and triglycerides, colostrum is an important energy source for heat production *via* diet-induced thermogenesis and non-shivering thermogenesis (14). This occurs thanks to the increased plasma triglyceride levels that BAT metabolizes. This effect was explored by Silva et al. (103), who observed that male Holstein calves that consumed an amount of colostrum equivalent to 15 to 20% of their body weight presented less shivering than those that received only 10%. This finding confirms that the metabolic thermogenic response is additional and long-term in nature, though authors like Clarke et al. (63) have stated that the response degree depends on exposure to cold. For example, in newborn lambs exposed to temperatures of 1°C, heat production by BAT involved around 80% of adipose tissue.

Therefore, producing heat of metabolic origin represents a long-term way to compensate for a drop in body temperature but comes at a high energy cost. This situation is related to the availability of energy resources at birth and the weight of newborns, conditions necessary for the proper functioning of their thermoregulatory mechanisms.

## Use of infrared thermography in assessing hypothermia in newborn ruminants

IRT systems have proven useful in assessing the thermal status of animals. The technique measures the radiation emitted from a body subject to changes in superficial microcirculation that increase or decrease these emissions (18, 97, 104–106). According to Villanueva-García et al. (105), the vasomotor effect caused by hypothermic states can be evaluated indirectly by tissue heat radiation, quantified by IRT. When peripheral circulation decreases, the heat radiated in some regions is also reduced. For the water buffalo, there is information on the application of IRT to assess thermoregulatory capacity under thermal stress conditions (17, 107). However, data of this kind have not been reported on newborn animals to analyze the mechanisms associated with temperature changes in extrauterine environments.

**Figure 5:**
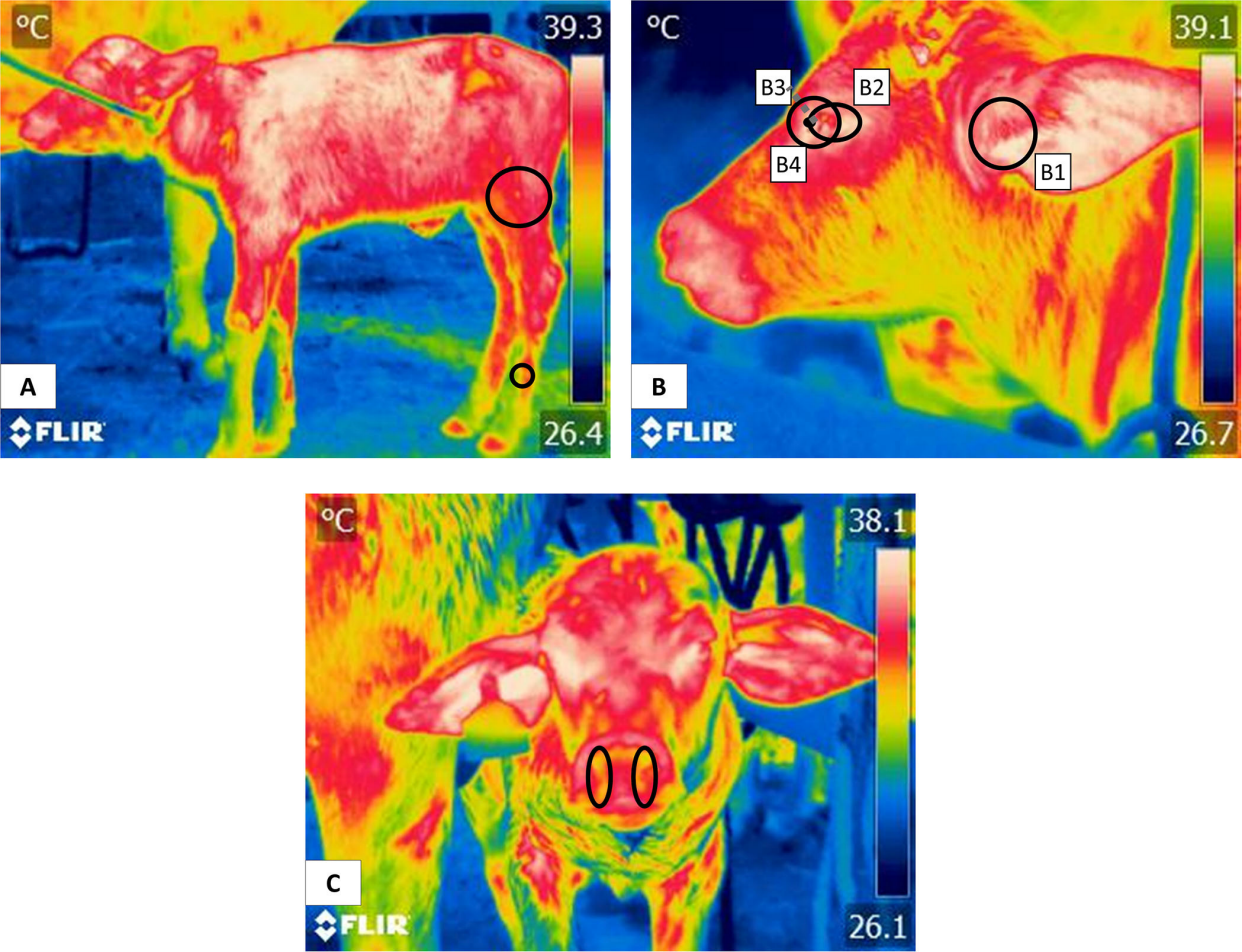
Thermal windows in newborn water buffalo. **(A)** pelvic limb, **(B)** facial window, **(B1)** auditory canal, **(B2)** ocular region, **(B3)** lacrimal gland, **(B4)** lacrimal caruncle, and **(C)** nostrils.

Evaluating degrees of hypothermia with IRT has been recommended on many occasions. However, authors caution that obtaining valid, reliable temperature values requires testing anatomical regions. For example, the auricular region is widely used in several ruminant and non-ruminant species to assess their thermal state (17, 108, 109) although concave surfaces are less likely influenced by external factors and may show a higher body surface temperature, while convex areas have the opposite effect, as shown in horses when measuring IRT in body regions such as elbow joint or the croup (110, 111). Likewise, specific key characteristics such as a high density of arteriovenous anastomoses, blood capillaries, and scarce –or no– hair or coat so that heat exchange with the environment can proceed through changes in the caliber of blood vessels (70, 112, 113). Figure 5 shows various thermal windows in newborn water buffaloes proposed in a preliminary study: (A) Pelvic limb. The windows in the pelvic limb are mainly in the femoral region, indicated by a circle that encompasses the cranial area at the level of the insertion of the quadriceps femoris and the tensor fascia lata, muscles irrigated by the femoral artery. At the distal level in the metatarsal region, the thermal response offered by the dorsal metatarsal artery is indicated with a circle. (B) Thermal facial windows. The windows of the auditory canal (B1), ocular region (B2), lacrimal gland (B3), and lacrimal caruncle (B4) are shown. Each window is delimited by regions like the auditory canal that, in turn, is delimited by the auricular folds of the auditory meatus. This ocular window is bounded to the upper and lower eyelids. The lacrimal caruncle window is delimited in the region of the medial canthus of the eye and allows the temperature evaluation that comes from the circulation of the infraorbital artery. (C) Nostrils. This window is delimited by the nostrils and allows its temperature evaluation together with the circulation of the maxillary artery that irrigates the area. Mota-Rojas et al. (17) described these regions and affirmed that in this species, the thermal windows of the lacrimal caruncle, ocular caruncle and corporals make it possible to assess thermal states.

Labeur et al. (114) evaluated the hip and back areas of 15 pregnant ewes grouped into two categories: sheared and unsheared. Both groups were exposed to a cold test for 1 hour. Findings showed that the temperature in the shoulder region was lower than in the hip and back areas. This difference could be related to adipose tissue deposits in these regions that constitute a resource for producing metabolic heat with no need for muscle contraction. Studies of this kind show that IRT could provide a way not only to assess levels of thermal comfort through the vascular reactions produced by cold responses but also to verify that regions with BAT deposits can cause increases in regional temperatures that reflect an organism's attempts to compensate for a temperature decrease and return to a thermoneutral state (115, 116). This coincides with the interscapular thermal window observations and BAT in laboratory animals (97).

**Figure 6:**
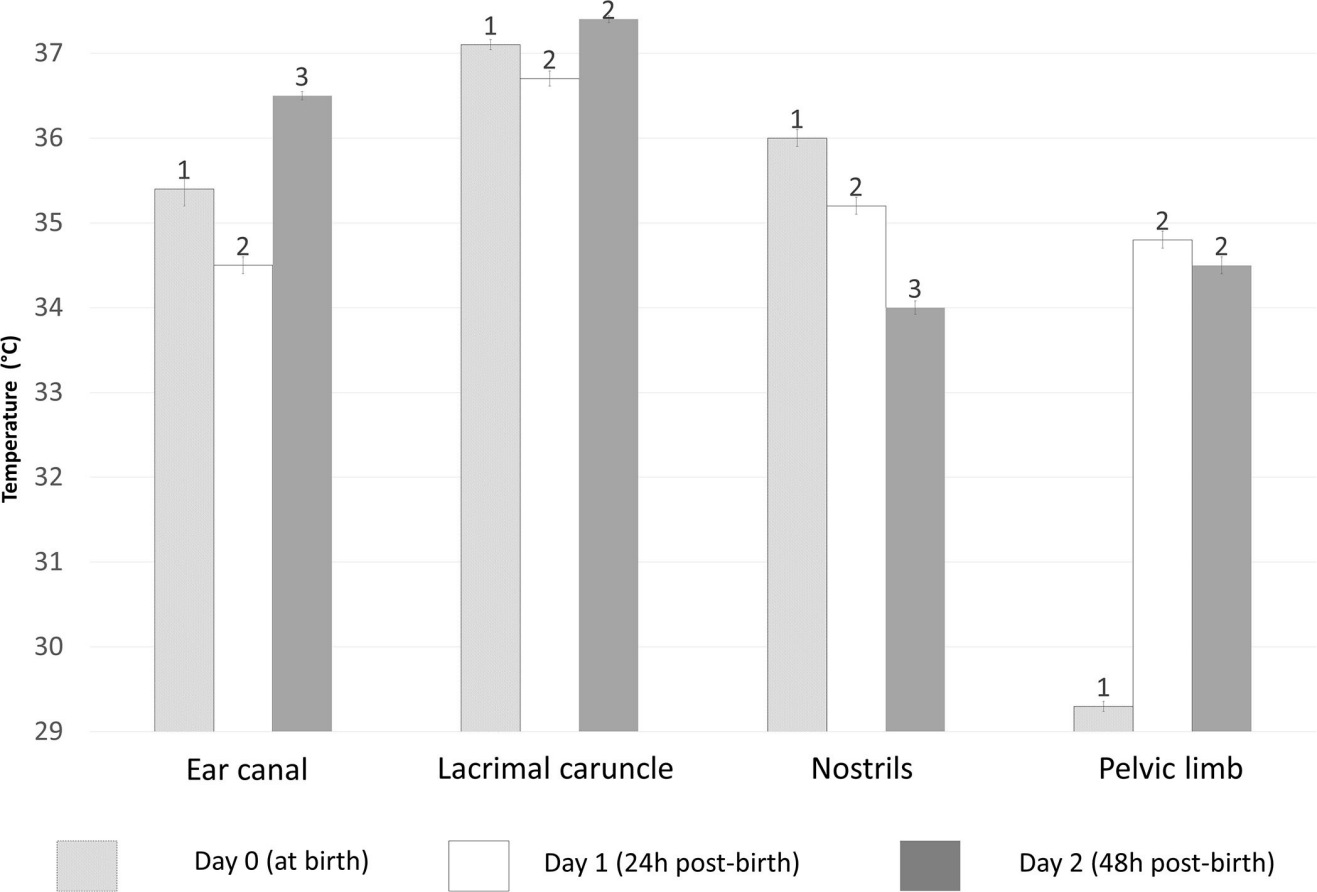
Effect of age measured in hours (0–48 h) on the temperature of newborn buffaloes in different thermal windows. In all events (hours), statistically significant differences (*p* < 0.0001) were observed in all measured times, except on the lacrimal gland and pelvic limb windows at 24 and 48 h post-birth. The average temperature at the ear canal differed from day 0 to day 2, with the highest value at 48 h post-birth (36.5°C) and the lowest at 24 h post-birth (34.5°C). The lacrimal gland window only showed differences of an average of 0.4°C from day 0 to day 1. The temperature of the nostril window showed a tendency to drop 0.8°C from birth to 24 h post-birth and 1.2°C from 24 h to 48 h post-birth. Meanwhile, a generally lower temperature was observed in the pelvic limb at birth (29.3°C) but it rose by 5.5°C at 24 h post-birth before decreasing by 0.3°C at 48 h post-birth. Data was obtained using variance analysis (ANOVA) by the mixed general linear model, with multiple comparisons of means using Tukey's test, Shapiro-Wilk normality test, and a significance level of *p* < 0.05. ^1, 2, 3^ Different numerals indicate significant differences between events according to the age of the buffalo calves (*p* < 0.05).

The responses measured in various thermal windows differ. Figure 6 shows thermal responses due to the effect of age determined during a preliminary study of neonatal water buffaloes (n = 50) conducted by the authors. Note that while the temperature of the lacrimal gland remained constant during parturition and at 24 h and 48 h of extrauterine life, that of the lower limbs showed greater temperature variation during the 2-day period. In the same thermal window the lowest temperatures were found in the thermal windows farthest from the body core. This can be explained by understanding the central and peripheral circulation of the organism. The vital structures in which important metabolic functions are carried out are found in the central parts of the body (17, 18), meaning the heart, lungs, brain, stomach, and kidneys. To protect these systems, when exposed to cold environments or situations that promote heat loss (such as the parturition process and their wet coat), the organism shifts blood supplies from peripheral structures to vital organs (72). Vasoconstriction at peripheral areas such as the thoracic and pelvic limbs, nose and tail prevents heat dissipation and also reduces said structures' superficial temperature (26). The increase in the temperature of the pelvic limb and nostril window according to the age of the newborn, could be explained because the newborn gets dry, reducing heat loss by evaporation, so the calf can reach a thermostability after drying the coat (1, 15) and after colostrum intake provides them of glucose and energy reserves (55).

The above brings us back to discussing the possible influence of environmental conditions and the season when birthing occurs. A study by Deak et al. (117) reported the effect of season and the physiological state of several body areas in 24 Holstein cows evaluated in the rainy (October- March) and dry seasons (April- September). They observed that temperatures in the flanks, perineal area, and lateral udder were 1.1°C higher in the rainy season than in the dry season and that temperatures of the udder, perineal, and rectal regions differed in the reproductive phase. These results suggest that heat loss or gain pathways are closely related to the seasonand may support the hypothesis that the survival of water buffalo neonates can vary with this factor (113, 118).

De Souza et al. (119) studied 23 Zebu × Holstein crossbred calves to evaluate whether cutaneous evaporative thermolysis occurs heterogeneously on the body surface of ruminants. Using IRT, they assessed the animals' limbs, neck, thorax, abdomen, and back under ambient temperatures <25°C, 25–29°C, and >29°C. Findings showed that cutaneous evaporative thermolysis did not differ among those regions but that temperatures in the neck had the highest thermal ranges. They also found that exposure to temperatures >29°C did not significantly increase evaporative thermolysis. Although those results confirm that heat loss is homogeneous throughout the body, some regions may show slight differences due to the arrangement of the blood vessels. This finding may be related to such features as skin thickness or a high density of hair follicles that can impede heat loss, though differences also occurred in the regions that allow heat loss (15, 113). Hristov et al. (120) evaluated the efficiency of IRT for determining temperatures in calves. Their work compared reference regions in the chest, shoulders, and legs to rectal temperatures and respiratory rates. They found that temperatures in the shoulders and chest had correlations of *r* = 0.9 and *r* = 0.65 with rectal temperature and respiratory rate, respectively, demonstrating that peripheral regions of ruminants respond to core body temperatures. This finding may help us better understand the buffalo as a model of precocial species.

In conclusion, IRT is a valuable tool that can be applied to recognize hypothermia in newborn ruminants. There is ample opportunity for its use in sheep, goats, cattle, and water buffaloes due to the limited information on this species. However, there is also a clear need for additional information on the effectiveness and reliability of different thermal windows and their capacity to indicate the ability of newborns to reestablish thermoneutrality.

## Perspectives

Although thermoregulation capacity in newborn ruminants such as lambs, kid goats, and calves can be similar to precocial species, some studies show anatomical-physiological differences between these species. For example, it is widely recognized that sheep have the greatest distribution of BAT in the hips and the interscapular region, which this species can use for heat production through NST (114). In contrast, kid goats have more type II muscle fibers in the neonatal stage than adults (49).

To date, it is not yet clear whether these differences could confer an advantage in some species as might be expected in large ruminants, where it is suggested that the presence of large muscle masses improves heat production by shivering. Species like the water buffalo and its limited available literature make them a potential field for future research within these animals. The water buffalo is a species recently introduced into production in numerous areas, so there are large areas of opportunity for research on the newborns of this species since we lack knowledge on the efficiency of their thermoregulation mechanisms (37). Reports mention mortality rates for water buffalo calves in Pakistan that range from 0.09% (121) to 7.59 ± 13.49% (122) and contrast to figures reported for domestic cattle (0.6–9.2%) (36, 37). However, factors such as season have been associated with variations in mortality rates, indicating that more neonatal casualties are observed in winter and spring (123), possibly due to low temperatures and increased rainfall. Likewise, because this species is being introduced into regions with extreme climates, mortality rates could be closely related to climatological phenomena, as observed in the variability in its thermal responses (124). Therefore, the study of the thermoregulatory mechanisms in these animals and breeding strategies could prevent hypothermia or, where appropriate, counteract this condition in this species.

BAT is another relevant study area in these species due to the differences in its distribution. Some authors have found that the degree of BAT presentation may be related to the expression of genes responsible for BAT differentiation and its deposit in regions such as the back (125). Similarly, the excessive deposit of BAT is related to the nutritional status of the dam. Therefore, designing strategies aimed at the mother's nutrition and, consequently, the body condition and weight at birth of the fetus could increase the effectiveness of the thermoregulatory mechanisms of newborn ruminants and their survival (126).

Within the thermoregulatory system of newborns, the activation of peripheral thermoreceptors such as transient potential receptors (TPR) is the first step to initiating every thermal response. The key role of these receptors, particularly TRPA1, TRPM8, and TRPC5 are triggered by cold stimuli and promote the vasomotor changes or contraction of skeletal muscle (91). However, to date, the available data is focused on small rodents. Therefore, studies targeting these receptors are a wide area of opportunity to investigate the functionality of TRP in ruminants.

The concentration of endogenous energy sources in the form of liver is essential because it is the first metabolic pathway to produce heat (53). However, both muscle and liver glycogens are a limited resource in the postpartum period, and variable according to the pregnancy characteristics, such as malnutrition present in the mother, which generates hypoglycemia in the ewe fetus (55). In this sense, using the mentioned resource, when colostrum is not ingested in the first 24 h of life, could ensure constant plasma glucose levels in calves (53), increasing the newborn's ability to thermoregulate at calving adequately.

On the other hand, the behaviors and postural changes that most mammals develop during exposure to cold to avoid heat loss by transfer, such as grouping or approaching warm areas, are another relevant issue in ruminants (17). These behavioral changes are not evident in ruminants, and it is suggested that they may be due to the specific anatomical characteristics such as the presence of hair, wool, and the color of the coat, elements that prevent active loss of heat in cold environments (14, 16). Similarly, it is important to study whether the phenotypic characteristics of adults, such as hair length, skin thickness, or hair follicles, influence the thermoregulatory capacity and mechanisms of newborn ruminants, as observed in wildlife (127). As we have seen, precocial species like ruminants have certain traits that make them more or less well-adapted to cope with hypothermia. Anatomy-physiological differences among newborn ruminants constitute the physiological basis for the various thermoregulation mechanisms found in different ruminant species.

The application of IRT as a tool to assess thermal status in mammals has been described by different authors (1, 17, 18, 128). But it is relevant to note that in current agricultural settings, using a hand-held thermographic camera may not be plausible in most large production units. This failure could be resolved by the automatization and optimization of IRT technologies that could precisely inform about thermal changes (129). For example, Schaeffer et al. (130) automatically detected the onset of bovine respiratory disease (BRD) in 65 Herford X Angus calves by placing an IRT scanning station around a feedlot pen. The corresponding software controlled the camera, collected thermal data, analyzed the image, and stored the information in a database. With the daily automatic monitoring of the orbital area, every time the animal accessed the water station, the authors obtained an average temperature of 33.85 ± 0.66°C for all calves, while true positive (TP) and true negative BRD animals had average temperatures of 35.44 ± 0.58°C and 34.71 ± 0.57°C, respectively. Therefore, it was possible to automatically record a 1°C elevation in the temperature of TP calves during the experimental period. Likewise, Zaninelli et al. (131) analyzed cow udder health status automatically taken through thermographic images, obtaining a significant relation between udder surface temperature and somatic cell counts with a sensitivity of 78.6% and a specificity of 77.9%. Technologies based on algorithms to detect a specific region such as the eye or the cheek in calves help to determine the maximum temperature of the area and were validated by comparing its measurements with a manual camera. The images taken by the algorithm were highly correlated to the hand-held device. They showed correlations (R2) of 0.99 for the eye and between 0.85 and 0.90 for the cheek (132). These studies present the possibility to integrate these automated systems for a field application of IRT on commercial farms.

Finally, the potential for utilizing remote tools like IRT means that we must determine the effectiveness of the various thermal windows that have been described for large ruminants (17, 133). Although the ocular and auricular regions have been mentioned as areas that indicate body temperature, another promising option is the appendicular region since it has been shown to be more sensitive to hypothermia due to the vascular supply it receives (2, 18).

## Conclusions

To confront the adverse effects of hypothermia, precocial species, including ruminants, are born with anatomical, physiological, and morphological characteristics that allow them to maintain their body temperatures. For example, one of the primary mechanisms is vasoconstriction of peripheral blood vessels to reduce heat loss through radiation. However, prolonged vasoconstriction can induce cellular hypoxia and impact the outcome of the newborn.

On the other hand, neonates have BAT deposits that serve as a substrate to NST to produce heat and prevent a sudden drop in core temperature, especially evaporative loss due to their wet coats at birth. The amount of BAT depends on the species and anatomical site (e.g., goats in the perirenal and inguinal region and cattle in the perirenal and subscapular). Moreover, its activation requires energy resources to produce heat by NST. Therefore, they can have consequences, such as inducing anaerobic metabolism that can lead to a state of acidosis. Finally, shivering thermogenesis requires muscular development of type II fibers in metabolically active muscles such as semitendinosus and biceps femoris, and its thermogenesis needs high energy reserves. One of them is glucose, an element that is limited in newborn ruminants and predisposes them to fast hypoglycemia. For this reason, critical factors like birth weight, available energy resources, and prompt colostrum ingestion need to be considered to prevent cases of hypothermia.

All these changes in microcirculation or metabolic activation of adipose tissue and their reflection of the superficial temperature of ruminant newborns allow the use of IRT as a valuable tool for recognizing hypothermia or identifying critical temperature drops during the first hours of life. The implementation of IRT on determining thermal windows such as ocular, auricular, flank, limbs, and neck, among others, have been shown to provide crucial information on the thermal status of newborn and the amount of heat radiation. However, additional validation is required to determine which ones are most appropriate for evaluation as a complementary step to installing preventive practices in ruminant neonates. Although, it should be noted that the lack of information on the application of IRT in newborn ruminants is a limitation and future studies may focus on this aspect.

## Author contributions

All authors contributed to the conceptualization, writing, reading, and approval of the final manuscript.

## Conflict of interest

The authors declare that the research was conducted in the absence of any commercial or financial relationships that could be construed as a potential conflict of interest.

## Publisher's note

All claims expressed in this article are solely those of the authors and do not necessarily represent those of their affiliated organizations, or those of the publisher, the editors and the reviewers. Any product that may be evaluated in this article, or claim that may be made by its manufacturer, is not guaranteed or endorsed by the publisher.
